# Poorly Differentiated Squamous Cell Carcinoma Arising in Tattooed Skin

**DOI:** 10.1155/2010/431813

**Published:** 2011-01-13

**Authors:** Deba P. Sarma, Renee B. Dentlinger, Amanda M. Forystek, Todd Stevens, Christopher Huerter

**Affiliations:** ^1^Department of Pathology, Creighton University Medical Center, Omaha, NE 68131, USA; ^2^Department of Dermatology, Creighton University Medical Center, Omaha, NE 68131, USA

## Abstract

*Introduction*. Tattoos have increasingly become accepted by mainstream Western society. As a result, the incidence of tattoo-associated dermatoses is on the rise. The presence of a poorly differentiated squamous cell carcinoma in an old tattooed skin is of interest as it has not been previously documented. *Case Presentation*. A 79-year-old white homeless man of European descent presented to the dermatology clinic with a painless raised nodule on his left forearm arising in a tattooed area. A biopsy of the lesion revealed a poorly differentiated squamous cell carcinoma infiltrating into a tattoo. The lesion was completely excised and the patient remains disease-free one year later. *Conclusion*. All previous reports of squamous cell carcinomas arising in tattoos have been well-differentiated low-grade type or keratoacanthoma-type and are considered to be coincidental rather than related to any carcinogenic effect of the tattoo pigments. Tattoo-associated poorly differentiated invasive carcinoma appears to be extremely rare.

## 1. Case Presentation

A 79-year-old white homeless male of European descent presented to the dermatology clinic complaining of a painless nodule on his left forearm arising in a tattooed area. The 5-cm black tattoo had been placed more than 50 years earlier in the mid 1950s without complications. The patient's medical history did not reveal any prior neoplastic disorders and was significant only for chronic alcohol abuse. 

Physical examination revealed a raised, 1-cm nonulcerated skin nodule surrounded by a large black tattoo. There were no palpable lymph nodes in the axilla.

A 4-mm punch biopsy was performed that revealed a poorly differentiated invasive squamous cell carcinoma. The tumor was completely excised with clear margins.

**Figure 1:**
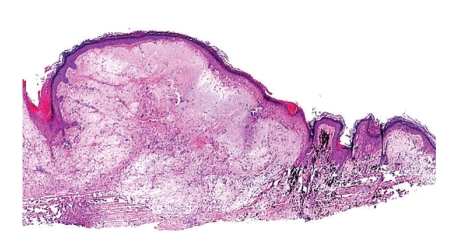
Low-power examination revealed abnormal keratin, severe dermal sun damage, tattoo pigment, and a cellular dermal neoplasm.

**Figure 2:**
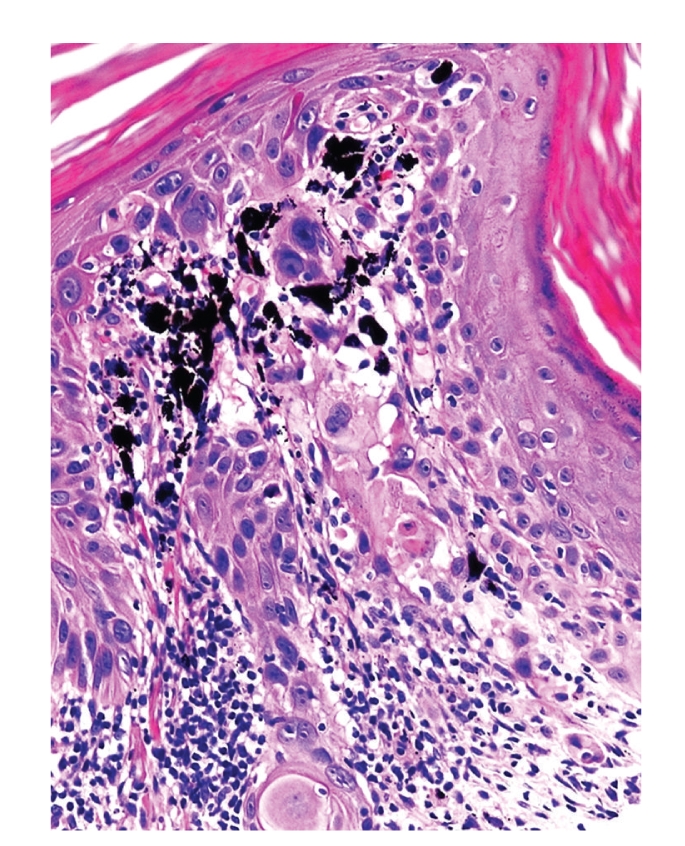
Basal keratinocyte dysplasia showing abnormal downward growth, abnormal surface keratin, and tattoo pigment within papillary dermis was noted.

**Figure 3:**
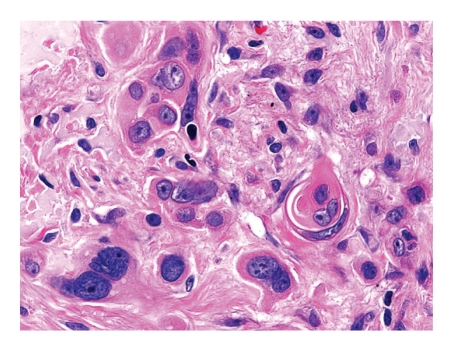
High-power photomicrograph demonstrates cells with enlarged nuclei, one-to-two prominent nucleoli, cytoplasmic keratin, well-defined cell borders, and early pearl formation, features indicative of squamous differentiation.

**Figure 4:**
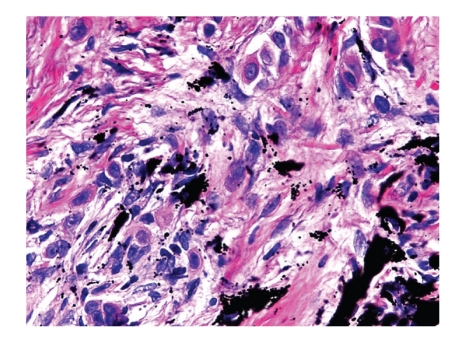
Epithelioid and spindled squamous cells are seen infiltrating amongst dermal tattoo pigment.

Microscopically, a poorly differentiated carcinoma was present within a sun-damaged dermis, infiltrating amongst dermal tattoo pigment as single cells and nests (Figures 1–4). Cytologically, the malignant cells displayed enlarged nuclei, one to two prominent nucleoli, abnormal cytoplasmic keratin, and intercellular bridges (desmosomes), typical of squamous differentiation (Figure 3). The overlying epidermis showed focal abnormal keratin and basal keratinocyte dysplasia (Figure 2). 

One year later, the patient remains free of any recurrence or metastasis.

## 2. Discussion

Once popular only in certain subcultures, tattoos have increasingly become accepted by mainstream Western society. As a result, the incidence of tattoo-associated dermatoses is on the rise. In a 2002 review of the literature, Jacobs divides dermatological reactions related to tattoos into three categories: allergic/granulomatous/lichenoid, inoculation/infection, and coincidental lesions [1]. Carcinomas within tattooed areas fall into the category of coincidental lesions. To date, all reports of squamous cell carcinoma (SCC) arising from tattoos have been well-differentiated or keratoacanthoma/SCC, keratoacanthoma type [2–7]. Such tumors are usually non aggressive. Of note, keratoacanthomas and keratoacanthoma type SCCs are considered to be the same lesion [5, 8, 9]. Most of the reported cases of tattoo-associated keratoacanthomas and keratoacanthoma type SCCs have occurred within one year after placement of the tattoo [2, 4, 5, 10], most of them related to red tattoo ink [5]. We found only one reported case of invasive well-differentiated SCC occurring 10 years after a tattoo placement in a 35-year-old man [4]. Our present case appears to be very different than those reported in the literature both clinically and histologically. Our 79-year-old patient developed a poorly differentiated squamous cell carcinoma in a black-tattooed skin more than 50 years after getting the tattoo.

Clinically or microscopically, there is little similarity between our case of poorly differentiated invasive SCC and the previously reported tattoo-related keratoacanthoma or keratoacanthoma type SCCs. Of course, our case may be a purely coincidental SCC arising in the sun-exposed and tattooed skin of the forearm of our elderly patient. 

The growing popularity of tattoos may be the result of increased social acceptance, cosmetic appearance, or the advent of effective laser removal. Significant risk factors for SCC include exposure to chronic ultraviolet exposure via sunlight or tanning beds, a tendency to burn rather than tan with sun exposure, overexposure or long-term exposure to ionizing radiation or laser treatment, exposure to carcinogenic or toxic compounds, and genetics. SCC generally presents in sun-exposed areas as a painless growth. Prior reports of SCC arising within tattooed areas have consisted entirely of well-differentiated lesions. To our knowledge, this is the first report of a poorly differentiated SCC found within a tattoo. 

Treatment of suspicious lesions should begin with a full thickness biopsy. If a microscopic diagnosis of SCC is confirmed, a complete surgical excision with clear margins is warranted. Subsequently, routine surveillance and careful followup is recommended as recurrence is possible. With complete excision, however, prognosis is favorable [7].

## 3. Conclusion

We encourage clinicians and pathologists to be aware of the possibility of malignant lesions arising within tattooed areas. With time, an accumulation of case reports will allow us to ascertain whether these lesions are in fact coincidental or attributable to a particular characteristic of the tattooing process [11]. As the number of tattoos placed continues to rise, so will the associated dermatoses, malignant or otherwise.
